# Three-Times Daily Ultrafractionated Radiation Therapy, A Novel and Promising Regimen for Glioblastoma Patients

**DOI:** 10.3390/cancers5041199

**Published:** 2013-09-25

**Authors:** Patrick Beauchesne

**Affiliations:** Neuro-Oncology Department, CHU de Nancy, Hospital Central, Nancy 54035, France; E-Mail: beauchesnep@wanadoo.fr; beaupatt@orange.fr; Tel.: +33-3-838-516-88; Fax: +33-3-838-527-34

**Keywords:** glioblastoma, radiotherapy, low-dose radiation therapy, ultra fractionated regimen

## Abstract

Glioblastomas are considered to be one of the most radio resistant tumors. Despite new therapies, the prognosis of this disease remains dismal. Also, the mechanisms of radiation resistance in mammalian cells are more complex than once believed. Experimental studies have indicated that some human cell lines are sensitive to low radiation doses of <1 Gy. This phenomenon has been termed low-dose hyper-radio-sensitivity (HRS), and is more apparent in radio resistant cell lines, such as glioblastoma cells. Sensitivity may result from the inability of low dose radiation to efficiently induce repair mechanisms, whereas higher doses cause enough damage to trigger repair responses for radio resistance. *In vitro* studies have demonstrated this phenomenon using various human malignant glioma cell lines: (1) daily repeated irradiation of cells with low doses compared to irradiation using a single biologically equivalent dose resulted in significantly higher cell killing; (2) experiments conducted on glioma xenografts demonstrated that repeated irradiation with low doses was more effective for inhibiting tumor growth than a single dose. In order to confirm and validate these promising studies on HRS, a few phase II trials were developed. For translating the experimental observations into the clinic, ultra fractionation protocols (with three daily doses) were tested in glioblastoma patients. Tolerance and toxicity were the primary endpoints, with overall survival as a secondary endpoint. These protocols were initiated before concomitant radio chemotherapy became the standard of care. For these trials, patients with an unfavorable clinical prognostic factor of newly unresectable GBM were included. When comparing the results of these trials with international literature using multivariate analysis for both progression free survival and overall survival, ultra fractionated irradiation showed superiority over radiotherapy alone. In addition, it was found to be equivalent to treatment using radiotherapy and temozolomide. Therefore, ultra fractionated protocols may prolong survival of glioblastoma patients. In this review, we describe the main experimental data regarding low-dose hypersensitivity as well as the findings of clinical trials that have investigated this new radiotherapy regimen.

## 1. Introduction

Malignant gliomas account for approximately 60% of all primary brain tumors [1,2]. Glioblastoma (GBM) is the most aggressive type of primary brain tumor in adults and is characterized by a high rate of local recurrence due to intrinsic radioresistance [1,2]. The prognosis of GBM patients remains dismal, and the reported median overall survival (OS) rarely exceeds 12 months [1,2,3]. Treatment consists of neurosurgical resection to the maximal feasible extent, which is followed by combined conformal brain radiotherapy and adjuvant chemotherapy using temozolomide (TMZ) when possible [1,2,3,4]. In a randomized trial conducted by the European Organization for Research and Treatment of Cancer (EORTC) and the National Cancer Institute of Canada (NCIC), Stupp et al. showed that GBM patients who received adjuvant radio chemotherapy (TMZ) followed by six courses of TMZ had a better survival compared to patients receiving adjuvant radiotherapy alone [4]. In fact, a significant increase in OS was observed in the radio chemotherapy group compared to the radiotherapy alone group (respective survival rates: 14.6 and 12.1 months).

Conformal radiation therapy remains the backbone of care for GBM. The target for irradiation is usually the tumor bulk, as visualized on cranial magnetic resonance imaging (MRI) with a wide margin of 2–3 cm. Although radiotherapy is not a curative treatment for GBM, it results in longer survival and optimized quality of life [1,2,3]. It is unclear whether clinical radio resistance in GBM is a result of intrinsic resistance at the cellular level. Therefore, future studies aimed at defining the molecular basis for radio resistance will be essential. The mechanisms involved in radiation resistance in mammalian cells are more complex than once believed. A few *in vitro* studies have shown that some human tumor cell lines can be sensitive to low radiation doses of <1 Gy, a phenomenon that has been termed low-dose hypersensitivity [5,6,7,8,9,10,11]. Strikingly, this “radio-sensitivity” is more apparent in radio resistant cell lines, such as glioma cells [5,6,7,8,9,10,11].

In this review, we discuss the 3-times daily ultra fractionated irradiation regimen for GBM. We not only describe the principal experimental results on low-dose hypersensitivity, but also report findings from phase II trials that have assessed ultra fractionated irradiation protocols in the clinic.

## 2. Experimental Studies

### 2.1. *In Vitro* Studies

**Figure 1:**
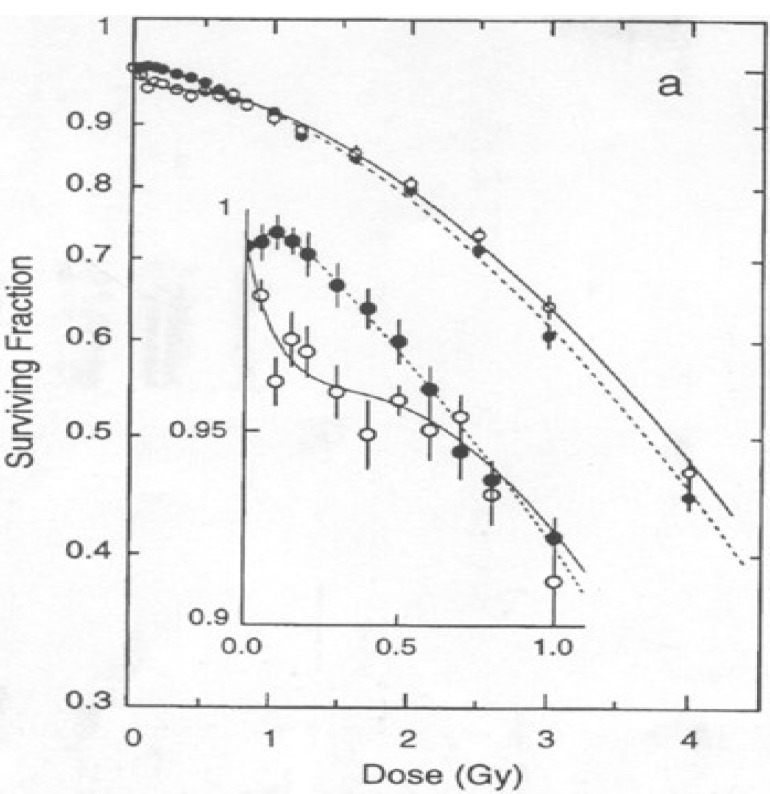
Low-dose hypersensitivity was represented as an undeniable downward “kink” on survival curve for doses below 1 Gy, followed for doses superior to 2 Gy by “IRR” or “increased radio-resistance” phenomenon.

The Gray Laboratory for Cancer Research was the first to identify increased X-ray sensitivity following very low doses per fraction in murine skin and kidney lines [5,6,7,8,9,10,11]. The V79 murine fibroblast line was irradiated at low doses, and cell survival was measured using a Dynamic Microscopic Imaging Processing Scanner (DMIPS) [5,6,7,8,9,10,11]. The results revealed hypersensitivity after very small doses (<0.3 Gy), followed by an enhancement in survival when the doses were increased (0.3–1 Gy) [5,6,7,8,9,10,11]. Wouters *et al*. confirmed the hyper-radio-sensitivity (HRS) phenomenon using a flow cytometry (FC)-based method for measuring survival, verifying that HRS was not merely an artifact associated with the DMIPS assay [5,6,7,8,9,10,11]. HRS was also observed in a human lung epithelial cell line (L132) as well as a Chinese hamster cell line [5,6,7,8,9,10,11]. Low-dose hypersensitivity could be seen on survival curves as an undeniable downward “kink” for doses <1 Gy, whereas doses >2 Gy increased the radio resistance phenomenon “IRR” [5,6,7,8,9,10,11] (Figure 1). Although it might be expected that the Linear Quadratic model could underestimate the amount of HRS, it seems to correlate with the data for doses ranging from 2 to 5 Gy [5,6,7,8,9,10,11].

While Lambin *et al*. demonstrated HRS in a human colorectal tumor cell line (HT 29) treated with low radiation doses, they failed to observe HRS in the same cell line after neutron irradiation at a dose rate of 0–20 Gy/min [12,13]. Furthermore, in another study, Lambin *et al*. irradiated several human tumor cell lines, including MeWo derived from melanoma, SW 48 from a colorectal tumor, and HX 142 from a neuroblastoma (considered radiosensitive) using low doses of radiation; however, they did find HRS (surviving fraction [SF] at 2 Gy ranging from 3 to 29%), [14]. Nevertheless, the failure to observe HRS in radio-sensitive cell line, HX 142, could be explained by decreased thresholds for inducible repair responses [14].

Among the radio-resistant tumors, GBM is considered as one of the most resistant. For this reason, Short *et al*. investigated the affect of low-dose irradiation on five human GBM cell lines (T98G, A7, U87MG, U138, and HGL21) and one anaplastic astrocytoma-derived cell line (U373) [15,16]. Survival time was calculated using the DMIPS method for T98G, A7, and U373, whereas the FC method (as modified by Wouters *et al*.) was utilized for U87MG, U138, and HGL21 [15,16]. The authors were able to demonstrate the HRS phenomenon in all five GBM cell lines, with the most dramatic results obtained for the A7, U138, and TG98G lines. However, U373 cells did not show HRS [15,16].

**Figure 2:**
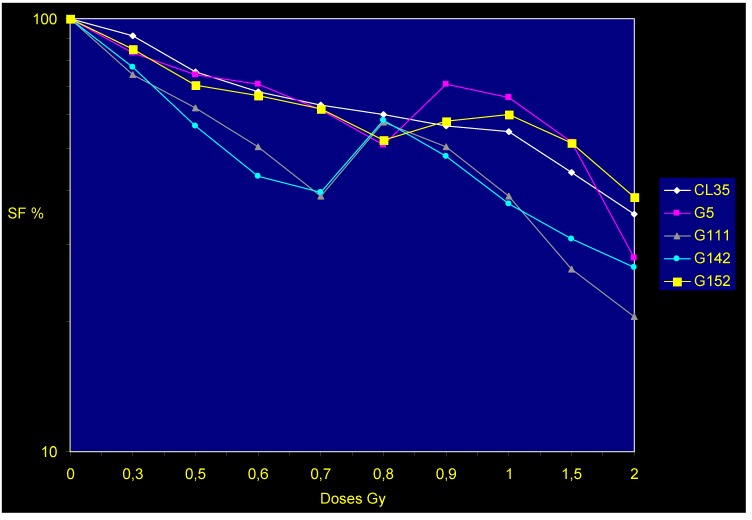
Survival of human glioma cells following irradiation. Cells were irradiated with 0–2 Gy. G5, G111, G142 and G152 glioma cell lines display HRS at doses below 1 Gy, except CL35.

Employing a linear accelerator that was routinely used on patients, a French team delivered low-dose radiation, which ranged from 0.2 to 2 Gy, to five human malignant glioma cell lines that were established in their laboratory (G5, CL35 [a clone from G5 cell line], G111, G142, and G152) [17]. Cell survival was calculated with the colony formation assay (CFA), and HRS was observed for doses <1 Gy for G5, G111, G142, and G152 cell lines; however, the sub-clone of G5 failed to show HRS [17] (Figure 2). Similarly, using doses <1 Gy, these same authors demonstrated HRS in four human melanoma tumor cell lines (M4Be, A375P, MeWo, and SKMe12) and MRC5 human fibroblasts, [17]. Through analysis of >26 different human cell lines, HRS had now been reported by several laboratories, which have used DMIPS and/or CFA to assess survival [12,13,14,15,16,17,18,19,20,21,22,23,24]. Taken together, HRS has been observed in colorectal carcinoma, bladder carcinoma, melanoma, prostate carcinoma, cervical squamous carcinoma, lung adenocarcinoma, neuroblastoma, and glioma cell lines, as well as a non-malignant lung epithelial line and a primary human fibroblast line [12,13,14,15,16,17,18,19,20,21,22,23,24].

Short *et al*. investigated the affect of repeated low radiation doses on the human T98G GBM cell line. In the study, cell survival was calculated by DMIPS after delivering 15 fractions of 0.4 Gy at 3 times a day (spaced by 4-h intervals) for five consecutive days, and the results were compared to the cumulative dose (1.2 Gy) given once daily for five successive days [25]. The repeated regimen led to a significant enhancement (*p* < 0.0002) in tumor cell killing, with lower cell survival occurring with the 3-times daily dose compared to the once-daily total dose [25]. Moreover, the authors treated two additional human GBM cell lines (A7 and U87) with repeated low doses, and found that the lowest cell survival occurred when doses were administered in intervals (*i.e.*, 4 and 6 h for A7 and 1 and 5 h for U87) [25]. Collectively, these experiments demonstrated that repeated low radiation doses could increase cell killing by enhancing the HRS phenomenon. This protocol of multiple low doses per fraction, per day, spaced at appropriate intervals (4 h), was termed “ultra fractionated regimen” [25].

Beauchesne *et al*. also studied the cumulative effect of low radiation doses on cell survival using the G5, CL35, and G152 GBM cell lines. For two consecutive days, three fractions of 0.8 Gy were spaced at 4-h intervals and compared to a single dose of 2 Gy. The ultra fractionated regimen produced a marked increase in cell killing in both G5 and G152 cell lines, but not in the CL35 line [17,26]. The irradiations were administered with a linear accelerator that was used daily for clinical patient therapies [17,26].

### 2.2. *In Vivo* Studies

Beck-Bornholdt *et al*. described the first study testing ultra fractionated irradiation in an animal model, which involved use of the rat rhabdomyosarcoma R1H, and 126 fractions of radiation over six weeks [27]. Notably, top-up irradiations were not given and different doses per fraction were applied (between 0.43 and 0.71 Gy) [27]. The results were compared to a “historical control”, and the authors found that the ultra fractionated regimen was slightly more effective than the conventional approach [27].

Krause *et al*. also developed an animal model. The A7 cell line, which displays HRS, was transplanted into the right hind legs of mice, and the irradiation protocol was initiated when the mean diameter of tumors reached 5 mm (a volume of 57 mm^3^) [28]. The ultra fractionated irradiation consisted of 126 fractions over six weeks (0.4 Gy per fraction; 3 fractions per day; 21 fractions per week; 6-h intervals), whereas the conventional treatment consisted of 30 fractions over six weeks (1.68 Gy per fraction; once daily; five fractions per week). A local irradiator was used with a dose rate of 0.2–0.4 Gy/min [28]. Surprisingly, it was found that the ultra fractionated regimen was less effective than the conventional regimen. The growth delay was significantly shorter in ultra fractionated irradiation compared to standard radiation (*p* = 0.047) [28].

**Figure 3:**
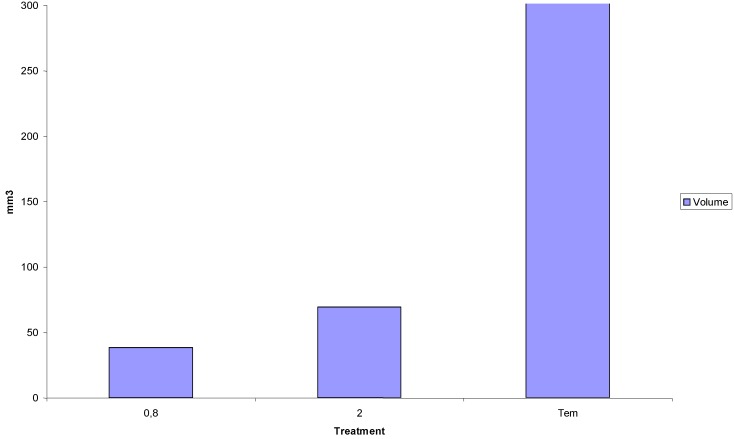
Inhibition of glioma tumor growth following repeated irradiation with low doses. G152 glioma cells injected into the interscapular region of 4-week-old female nude mice (Swiss nu/nu). Seventeen days after grafting, mice were exposed either to 0.8 Gy 3-times/day spaced by 4 h 5 days/week for two consecutive weeks or to 2 Gy once/day 5 days/week for two consecutive weeks.

In addition, Beauchesne *et al*. tested fractionated low-dose irradiation in a glioma animal model that was previously developed in their laboratory using the G152 cell line [17,26]. G152 xenograft tumors were grown for 17 days, and the mice were then exposed to either 0.8 Gy per fraction (3 times per day; spaced at 4-h intervals; four days per week; two consecutive weeks) or a single dose of 2 Gy (once per day; four days per week; two consecutive weeks) [17,26]. Irradiation was delivered by a clinical linear accelerator, and the mice were immobilized in plastic tubes so that only the tumor was exposed to the radiation [17,26]. The regimen of repeated low-doses had a therapeutic effect on tumor growth. In fact, at week 12, tumor volume in the ultra fractionated group was half that of the mice receiving standard therapy (*p* = 0.0022) [17,26] (Figure 3). Furthermore, a second experiment was conducted to compare irradiation regimens for the same total doses. For this part of the study, at 17 days post grafting, the mice were exposed to either 0.8 Gy 3 times per day (4-h intervals; five days per week; two consecutive weeks; total of 24 Gy) or to 2.4 Gy once daily (five days per week; two consecutive weeks; total of 24 Gy) [17,26]. As previously demonstrated, the ultra fractionated regimen resulted in dramatic inhibition on tumor growth. Importantly, comparing the two experimental conditions indicated that regardless of whether the mice were irradiated with 2-Gy or 2.4-Gy fractions, they displayed similar tumor growth [17,26]. Taken together, these experiments showed that ultra fractionated irradiation provided a marked benefit compared to classical irradiation regimens.

### 2.3. Mechanisms of HRS

The mechanisms underlying the cell-type specific expression of HRS are still being investigated, but appear related to defective DNA repair systems and cell cycle regulation [11]. HRS is more likely to affect early-responding proliferating tissues, such as skin, and Harney *et al*. have demonstrated a response consistent with HRS in human skin [29]. Clearly, more molecular-based experiments are needed using whole-animal models to characterize the mechanisms of HRS in normal tissue radiation injury. Clinical data obtained so far are also consistent with the concept of transitional low-dose radiation responses in tumor nodules derived from solid tumors [30].

### 2.4. Anti-Neoplastic Agents

It was of interest to study the combination of low radiation doses and few anti-neoplastic agents. Taxanes were used in combination to HRS on head and neck squamous cell carcinoma cell lines and on SCCHN tumor xenografts in nude mice., a significant enhancement of radiation sensitization by taxanes was observed [31,32]. Beauchesne *et al*. tested a combination of etoposide and HRS upon malignant glioma cell lines, low-dose hypersensitivity is apparent when etoposide is administrated immediately after irradiation, resulting in an additive effect on SF [17].

## 3. Clinical Studies

To translate these *in vitro* and *in vivo* observations into the clinical setting, a few phase II clinical studies were initiated for assessing ultra fractionated radiation regimens in patients.

### 3.1. *De Novo* Tumors

Beauchesne *et al*. initiated a phase II clinical study testing an ultra fractionated regimen that delivered a total dose of 67.5 Gy over 90 fractions (0.75-Gy fractions; 3-times daily; at least 4-h intervals; five fractions per week; six consecutive weeks) in newly-diagnosed, unresected supratentorial GBMs [25]. The objective of the study was to assess the toxicity and tolerance of the ultra fractionated regimen. Notably, this protocol was initiated before concomitant radio-chemotherapy became the standard of care for GBM patients. Also, this was a multi-center French study. Eligible patients were >18 years of age, gave informed consent, and were newly diagnosed with unresectable GBM with a World Health Organization (WHO) performance status of 0–2 [33]. Secondary end-points included progression-free survival (PFS) and OS. Irradiation was delivered to the gross tumor volume using a 2.5-cm margin for clinical target volume, and the radiation therapy was coordinated through dedicated computed tomography (CT) or magnetic resonance imaging (MRI) along with three-dimensional planning systems. Conformal ultra fractionated radiotherapy was delivered with linear accelerators with a nominal energy of ≥6 MeV [25]. Thirty-one patients were included (16 males and 15 females) with a median age of 58 years old and a median Karnofsky Performance Status of 80 [33]. Four patients died before initiating irradiation, and two decided to revert to standard radiation therapy. The ultra fractionated regimen was completed in 22 patients, and multi focal GBM was reported in seven patients [33]. No toxic death occurred during the ultra fractionated irradiation, and the most common adverse effect was fatigue, which is frequently associated with cranial radiation therapy. Although the ultra fractionation regimen was a constraint for patients, it was well tolerated. Two patients with very large tumors progressed during the radiotherapy, leading to premature discontinuation after 48 and 56 Gy [25]. Tumor response was analyzed, and eight stabilizations were reported. In most cases, corticosteroids were decreased or stopped for several weeks. The median overall survival was 9.53 months, and the overall survival rate at 18 and 24 months was 19.35% and 15.48%, respectively. Moreover, half of the long-term survivors did not receive a chemotherapy line. The median PFS was 5.09 months, and the PFS rate at six and 12 months was 45.16% and 12.90%, respectively [33].

**Figure 4:**
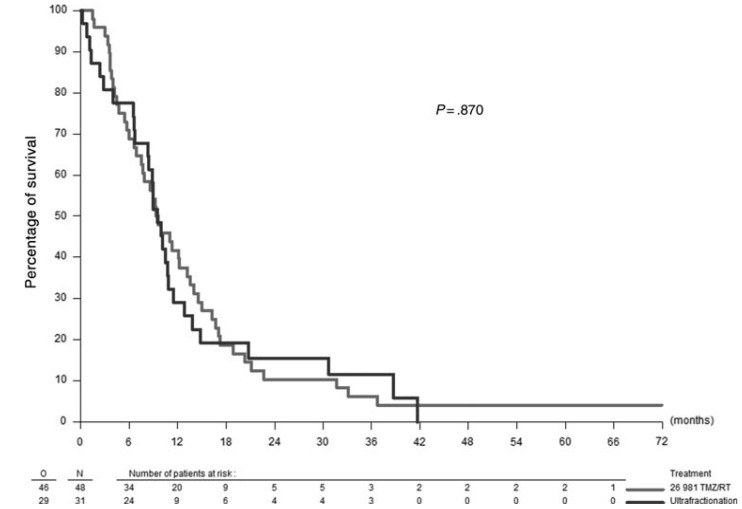
Overall survival: ultra-fractionated schedule *vs*. EORTC/NCIC temozolomide/radiation therapy.

This trial represented the first time that an ultra fractionated irradiation regimen was clinically performed and tested. The ultra fractionated regimen was safe and well tolerated. Notably, no post-irradiation leuco-encephalopathy was reported. In fact, the results achieved were comparable to the highest survival that had been achieved in recent randomized studies [4,34,35]. Also, it is important to point out that 16.1% of the patients included in the study were ≥70 years old. The EORTC–NCIC study reported a respective median overall survival of 7.85 and 9.4 months for irradiation alone and combined TMZ-radiation therapy in patients with unresectable GBM [4,26,29] (Figure 4). There was a marked number of long survivors that were reported with this low-dose radiation therapy, and the overall survival rate at 24 months was 15.48%, which compares favorably with data for TMZ–radiation therapy obtained in the EORTC–NCIC study (10.42%) [4].

These encouraging results supported the development of further clinical trials to investigate the efficacy of the ultra fractionated regimen in combination with adjuvant TMZ. Thus, Beauchesne *et al*. initiated a prospective multicenter phase II trial to assess the effectiveness ultra fractionated irradiation along with concomitant and adjuvant TMZ for treating *de novo* unresectable GBM [36]. Patients >18 years old, who were able to give informed consent, and had histologically proven newly diagnosed, inoperable supratentorial GBM were eligible [30]. Three doses of 0.75 Gy were delivered daily at a minimum of 4-h intervals (five days a week; six consecutive weeks), and concomitant chemotherapy consisting of TMZ was given seven days per week during the ultra fractionated radiation therapy. After a 4-week break, chemotherapy was resumed with up to six cycles of adjuvant TMZ every 28 days [36]. Irradiation was delivered to the gross tumor volume with a 2.5-cm margin for the clinical target volume, and radiation therapy was again coordinated using dedicated CT or MRI scanning along with three-dimensional planning systems. Conformal ultra fractionated radiotherapy was delivered using a linear accelerator with a nominal energy of ≥6 MeV. Tolerability and toxicity were the primary endpoints, whereas survival and PFS were the secondary endpoints. Forty-two patients were included (30 males and 12 females), with a median age of 58 (range: 29 to 77 years old) and median Karnofsky Performance Status of 80 [36]. All patients received combined ultra fractionated irradiation and TMZ. Three patients progressed during the scheduled radiation therapy, and four sudden deaths were reported [36]. No grade 3–4 CNS toxicity was noted; however, one case of grade 4 hematological toxicities was observed, and two cases of pulmonary infection were reported (all were fatal). Four complete responses were observed, and 12 patients showed a partial response (PR) and stabilization (S) [30]. The median OS was 15 months, and the OS rate at 18 and 24 months was 37% and 25.9%, respectively [36]. Eight patients were alive at the time of first analysis. These preliminary results have confirmed that this combination of ultra fractionated irradiation and TMZ is feasible, safe, and well tolerated. Moreover, encouraging and unexpected survival was noted in GBM patients with unfavorable features. The definitive analysis for this study is planned for the end of 2013. A molecular analysis is also scheduled.

### 3.2. Recurrent Tumors

Siker *et al*. conducted a phase I/II study to test on recurrent malignant glioma patients, a protracted semi continuous low dose rate radiotherapy, SLDR [37]. The delivery of SLDR is feasible in patients with recurrent gliomas and resulted in improved outcomes for patients who underwent re-resection., this work suggested that dose rate and not the pulse is the reason for the observed efficacy [37].

No clinical trial has been reported or developed for the treatment of recurrent GBM patients using HRS phenemenon. Only one case report was found in the literature. Jahraus *et al*. reported the case of an 82-year-old woman with GBM that progressed during standard treatment [38]. The woman completed radiotherapy plus concomitant TMZ, and recurrence occurred at the end of the treatment. It was then decided to modify the therapy, and the patient was given a combination of ultra fractionated irradiation (0.5 Gy, whole brain; twice daily; 4-h intervals) and chemotherapy, consisting of TMZ administered in a dose-intense regimen (5 days every 14-day cycle) [38]. This new treatment resulted in regression of the tumor recurrence as assessed by MRI, and the patient survived for approximately six months following recurrence, having received five cycles of additional modified therapy [38]. Therefore, this combination of TMZ and ultra fractionated irradiation was efficacious and well tolerated [38].

### 3.3. Pulse Reduced Dose Rate Radiotherapy

Pulsed reduced-dose-rate radiotherapy (PRDR) is a re irradiation technique that reduces the effective dose rate and increases the treatment time, allowing sub lethal damage repair during irradiation [39]. This pulse radiotherapy regimen was tested on intracranial U87MG GBM tumors in nude mice, irradiation consisted in ten 0.2-Gy pulses separated by 3-min intervals [39]. Pulse reduced regimen of radiation therapy resulted in greater inhibition of tumor growth and improved survival [39]. In another animal study, the efficacy of pulse reduced dose rate irradiation was monitored by micro PET, the results confirmed the previously data [40]. Translation to the clinic was performed. A man with a grade II astrocytoma who progressed to a GBM after surgery, radiation therapy and temozolomide, was re irradiated by a pulse reduce dose rate radiotherapy; a series of 0.2 Gy pulses separated by 3 min time intervals, creating an apparent dose rate of 0.0667 Gy/min [41]. A clinical response was obtained, and no apparent acute or late neurologic toxicities [41]. Adkison *et al*. treated 103 patients with recurrent primary central nervous system malignancies with pulse reduce dose rate radiotherapy; a series of 0.2-Gy pulses at 3-min intervals, creating an apparent dose rate of 0.0667 Gy/min to a median dose of 50 Gy (range, 20–60) delivered in 1.8–2.0-Gy fractions. [42]. Pulsed reduced dose rate was well tolerated, allowing for safe re treatment of larger target volumes to high doses with palliative benefit, and for Grade 4 patients at recurrence, an interval from initial RT of >14 months predicted for longer survival after pulse reduced dose rate radiotherapy [42].

## 4. Conclusions

The “HRS” phenomenon has been demonstrated *in vitro* using numerous human cancer cell lines, including malignant glioma lines. Interestingly, daily repeated irradiation of cells with low doses compared to irradiation with a single biologically equivalent dose resulted in significantly higher cell killing. Moreover, experiments conducted using glioma xenografts confirmed that low-dose ultra fractionated irradiation (0.8 Gy, three times a day) more effectively inhibited tumor growth than a single dose (2 or 2.4 Gy, once a day). Furthermore, clinical trials using ultra fractionated radiation regimens verified these experimental results and have proved that the treatment method is safe and well tolerated. When compared with the EORTC/NCIC trial results for both PFS and OS in multivariate analysis, ultra fractionation showed superiority over radiation therapy alone, but not over radio-chemotherapy (with TMZ). Moreover, the preliminary results of combined ultra fractionated irradiation along with concomitant or adjuvant TMZ yielded encouraging survival outcomes. Taken together, ultra fractionated irradiation seems to be a promising treatment for GBM patients.

## References

[1] Behin A., Hoang-Xuan K., Carpentier A.F., Delattre J.Y. (2003). Primary brain tumours in adults. Lancet.

[2] De Angelis L.M. (2001). Brain tumors. N. Engl. J. Med..

[3] Stewart L.A. (2002). Chemotherapy in adult high-grade glioma: A systematic review and meta-analysis of individual patient data from 12 randomised trials. Lancet.

[4] Stupp R., Mason W.P., van den Bent M.J., Weller M., Fisher B., Taphoorn M.J., Belanger K., Brandes A.A., Marosi C., Bogdahn U. (2005). Radiotherapy plus concomitant and adjuvant temozolomide for glioblastoma. N. Engl. J. Med..

[5] Joiner M.C., Denekamp J. (1986). The effect of small radiation doses on mouse skin. Br. J. Cancer.

[6] Joiner M.C., Denekamp J., Maughan R.L. (1986). The use of “top-up” experiments to investigate the effect of very small doses per fraction in mouse skin. Int. J. Radiat. Biol. Relat. Stud. Phys. Chem. Med..

[7] Joiner M.C., Johns H. (1988). Renal damage in the mouse: The response to very small doses per fraction. Radiat. Res..

[8] Marples B., Joiner M.C. (1993). The response of Chinese hamster V79 cells to low radiation doses: Evidence of enhanced sensitivity of the whole cell population. Radiat. Res..

[9] Joiner M.C., Marples B., Lambin P., Short S.C., Turessson I. (2001). Low-dose hypersensitivity: Current status and possible mechanisms. Int. J. Radiat. Oncol. Biol. Phys..

[10] Marples B., Collis S.J. (2008). Low-dose hyper-radiosensitivity: Past, present, and future. Int. J. Radiat. Oncol. Biol. Phys..

[11] Martin L.M., Marples B., Lynch T.H., Hollywood D., Marignol L. (2013). Exposure to low dose ionising radiation: Molecular and clinical consequences. Cancer Lett..

[12] Lambin P., Marples B., Fertil B., Joiner M.C. (1993). Hypersensitivity of a human tumour cell line to very low radiation doses. Int. J. Radiat. Biol..

[13] Lambin P., Malaise E.P., Joiner M.C. (1993). Megafractionnement: Une methode pour agir sur les tumeurs intrinsequement radioresistantes?. Bull. Cancer Radiother..

[14] Lambin P., Malaise E.P., Joiner M.C. (1996). Might intrinsic radioresistance of human tumour cells be induced by radiation?. Int. J. Radiat. Biol..

[15] Short S.C., Mayes C.R., Woodcock M., Joiner M.C. (1999). Low dose hypersensitivity in the T98G human glioblastoma cell line. Int. J. Radiat. Biol..

[16] Short S.C., Mitchell S.A., Boulton P., Joiner M.C. (1999). The response of human glioma cell lines to low-dose radiation exposure. Int. J. Radiat. Biol..

[17] Beauchesne P., Bertrand S., Branche R., Linke S.P., Revel R., Dore J.F., Pedeux R.M. (2003). Human malignant glioma cell lines are sensitive to low radiation doses. Int. J. Cancer.

[18] Joiner M.C., Marples B., Johns H. (1993). The response of tissues to very low doses per fraction: A reflection of induced repair?. Cancer Res..

[19] Joiner M.C., Lambin P., Malaise E.P., Robson T., Arrand J.E., Skov K.A., Marples B. (1996). Hypersensitivity to very-low single radiation doses: Its relationship to the adaptive response and induced radioresistance. Mutat. Res..

[20] Joiner M.C., Marples B., Lambin P., Short M.C., Turesson I. (2001). Low-dose hypersensitivity: Current status and possible mechanisms. Int. J. Radiat. Oncol. Biol. Phys..

[21] Lambin P., Skov K.A., Joiner M.C. (1997). Low dose hyper-radiosensitivity and increased radioresistance in mammalian cells. Int. J. Radiat. Biol..

[22] Singh B., Arrand J.E., Joiner M.C. (1994). Hypersensitive response of normal human lung epithelial cells at low radiation doses. Int. J. Radiat. Biol..

[23] Turesson I., Joiner M.C. (1996). Clinical evidence of hypersensitivity to low doses in radiotherapy. Radiother. Oncol..

[24] Wouters B.G., Sky A.M., Skarsgard L.D. (1996). Low dose hypersensitivity and increased radioresistance in a panel of human tumor cell lines with different radiosensitivity. Radiat. Res..

[25] Short S.C., Kelly J., Mayes C.R., Woodcock M., Joiner M.C. (2001). Low-dose hypersensitivity after fractionated low- dose irradiation *in vitro*. Int. J. Radiat. Biol..

[26] Pedeux R., Boniol M., Dore J.F., Beauchesne P. (2003). Ultrafractionation radiation therapy of human gliomas; a pre-clinical model. Int. J. Cancer.

[27] Beck-Bornholdt H.P., Maurer T., Becker S., Omniczynski M., Vogler H., Würschmidt F. (1989). Radiotherapy of the rhabdomyosarcoma R1H of the rat: Hyperfractionation–126 fractions applied within 6 weeks. Int. J. Radiat. Oncol. Biol. Phys..

[28] Krause M., Hessel F., Wohlfarth J., Zips D., Hoinkis C., Foest H., Petersen C., Short S.C., Joiner M.C., Baumann M. (2003). Ultrafractionation in A7 human malignant glioma in nude mice. Int. J. Cancer.

[29] Harney J., Short S.C., Shah N., Joiner M., Saunders M.I. (2004). Low dose hyper-radiosensitivity in metastatic tumors. Int. J. Radiat. Oncol. Biol. Phys..

[30] Harney J., Shah N., Short S.C., Daley F., Groom N., Wilson G.D., Joiner M.C., Saunders M.I. (2004). The evaluation of low dose hyper-radiosensitivity in normal human skin. Radiother. Oncol..

[31] Shajahan S., Brown B., Dey S., Lele S.M., Valentino J., Jones R., Mohiuddin M., Ahmed M.M., Spring P.M., Arnold S.M. (2004). Low dose fractionated radiation potentiates the effects of taxotere in nude mice xenografts of squamous cell carcinoma of head and neck. Cell Cycle.

[32] Dey S., Spring P.M., Arnold S., Valentino J., Chendil D., Regine W.F., Mohiuddin M., Ahmed M.M. (2003). Low-dose fractionated radiation potentiates the effects of Paclitaxel in wild-type and mutant p53 head and neck tumor cell lines. Clin. Cancer Res..

[33] Beauchesne P., Bernier V., Carnin C., Taillandier L., Djabri M., Martin L., Michel X., Maire J.P., Khalil T., Kerr C. (2010). Prolonged survival for patients with newly diagnosed, inoperable glioblastoma with 3-times daily ultrafractionated radiation therapy. Neuro-oncology.

[34] Athanassiou H., Synodinou M., Maragoudakis E., Paraskevaidis M., Verigos C., Misailidou D., Antonadou D., Saris G., Beroukas K., Karageorgis P. (2005). Randomized phase II study of temozolomide and radiotherapy compared with radiotherapy alone in newly diagnosed glioblastoma multiforme. J. Clin. Oncol..

[35] Westphal M., Hilt D.C., Bortey E., Delavault P., Olivares R., Warnke P.C., Whittle I.R., Jääskeläinen J., Ram Z. (2003). A phase 3 trial of local chemotherapy with biodegradable carmustine (BCNU) wafers (Gliadel wafers) in patients with primary malignant glioma. Neuro-oncology.

[36] Beauchesne P., Faure G., Noel G., Schmitt T., Martin L., Jadaud E., Carnin C.  (2011). TEMOFRAC—A phase II trial. concurrent 3-times daily ultrafractionated radiation therapy and temozolomide for newly inoperable glioblastomas. J. Clin. Oncol..

[37] Siker M.L., Firat S.Y., Mueller W., Krouwer H., Schultz C.J. (2012). Semicontinuous low-dose-rate teletherapy for the treatment of recurrent glial brain tumors: Final report of a phase I/II study. Int. J. Radiat. Oncol. Biol. Phys..

[38] Jahraus C.D., Friedman A.H. (2010). Chemopotentiation by unltrafractionated radiotherapy in glioblastoma resistant to conventional therapy. Tumori.

[39] Park S.S., Chunta J.L., Robertson J.M., Martinez A.A., Wong C.Y.O., Amin M., Wilson G.D., Marples B. (2011). MicroPET/CT imaging of an orthotopic model of human glioblastoma multiforme and evaluation of pulsed low-dose irradiation. Int. J. Radiat. Oncol. Biol. Phys..

[40] Dilworth J.T., Ktueger S.A., Dabian M., Grills I.S., Torma J., Wilson G.D., Marples B. (2013). Pulsed low-dose irradiation of orthotopic glioblastoma multiforme (GBM) in a pre-clinical model: Effects on vascularization and tumor control. Radiother. Oncol..

[41] Cannon G.M., Tomé W.A., Robins H.I., Howard S.P. (2007). Pulsed reduced dose-rate radiotherapy: Case report: A novel re-treatment strategy in the management of recurrent glioblastoma multiforme. J. Neurooncol..

[42] Adkison J.B., Tomé W., Seo S., Richards G.M., Robins H.I., Rassmussen K., Welsh J.S., Mahler P.A., Howard S.P. (2011). Reirradiation of large-volume recurrent glioma with pulsed reduced-dose-rate radiotherapy. Int. J. Radiat. Oncol. Biol. Phys..

